# Skeletal muscle mitochondrial impairment in patients with newly diagnosed multiple sclerosis revealed by ¹H/³¹P magnetic resonance spectroscopy

**DOI:** 10.1038/s41598-026-54705-8

**Published:** 2026-06-11

**Authors:** Branislav Kollár, Adela Penesová, Radka Klepochová, Pavel Šiarnik, Miroslav Vlček, Patrik Krumpolec, Lucia Mosná, Richard Imrich, Peter Turčáni, Žofia Rádiková, Martin Krššák

**Affiliations:** 1 1st Department of Neurology, Faculty of Medicine, Comenius University and University Hospital, Bratislava, Slovakia; 2 Institute of Clinical and Translational Research, Biomedical Research Center, Slovak Academy of Sciences, Bratislava, Slovakia; 3 Department of Biological and Medical Science, Faculty of Physical Education and Sport, Comenius University Bratislava, Bratislava, Slovakia; 4 Department of Biomedical Imaging and Image-Guided Therapy, High-Field MR Center, Medical University of Vienna, Vienna, Austria; 5 Institute of Measurement Science, Slovak Academy of Sciences, Bratislava, Slovakia; 6 Faculty of Medicine, Slovak Medical University in Bratislava, Bratislava, Slovakia; 7 Division of Endocrinology and Metabolism, Department of Internal Medicine III, Medical University of Vienna, Vienna, Austria; 8 Institute of Immunology, Faculty of Medicine, Comenius University Bratislava, Bratislava, Slovakia; 9 Faculty of Health Sciences, University of Ss. Cyril and Methodius in Trnava, Trnava, Slovakia

**Keywords:** Proton and phosphorus magnetic resonance spectroscopy, Multiple sclerosis, Skeletal muscle mitochondrial dysfunction, Biochemistry, Diseases, Endocrinology, Physiology

## Abstract

Mitochondrial dysfunction is implicated in the pathophysiology of multiple sclerosis (MS) and may contribute to its fatigue and muscle weakness. Whether skeletal muscle metabolic changes are present at disease onset is unclear. In this observational matched case-control study we investigated muscle metabolism and its relation to systemic metabolic measures in newly diagnosed, treatment-naïve patients with MS (PwMS) and matched healthy controls. Gastrocnemius metabolism was assessed using static and dynamic 7 T ^1^H/^31^P magnetic resonance spectroscopy (MRS), measuring energy metabolites, membrane-related compounds, and post-exercise phosphocreatine (PCr) recovery. Systemic metabolism was assessed by oral glucose tolerance test (OGTT) measuring glucose, insulin, and GLP-1. Static MRS revealed lower carnosine (*p* = 0.044) and a trend toward lower acetylcarnitine in PwMS. Dynamic ³¹P-MRS showed elevated pre-exercise inorganic phosphate (Pi; *p* = 0.012), trend toward reduced PCr/Pi ratio, and prolonged PCr recovery (*p* = 0.031) in PwMS. PwMS also had reduced GLP-1 response during OGTT (*p* = 0.032) despite preserved insulin sensitivity. Higher PCr levels were associated with greater GLP-1 response in PwMS only; longer PCr recovery was associated with insulin resistance parameters in controls only. Newly diagnosed MS is associated with skeletal muscle mitochondrial impairment during energetic stress, despite largely preserved systemic metabolic status. Altered metabolites, delayed recovery, and association with reduced GLP-1 response suggest impaired muscle bioenergetics and altered systemic metabolic regulation present at disease onset. Targeting mitochondrial and metabolic pathways offers therapeutic potential in early MS.

Trial registration number: ClinicalTrials.gov: NCT03052595, date of registration: 14th Feb 2017.

## Introduction

Multiple sclerosis (MS) is a chronic immune-mediated disease of the CNS, typically manifesting in young adults with heterogeneous motor, sensory, visual, and autonomic symptoms. MS is characterized by inflammation, demyelination, reactive gliosis, and axonal degeneration, leading to irreversible disability. Its etiopathogenesis is multifactorial, involving genetic, immunologic, and environmental factors^1^. Recent topics include immune mechanisms triggered by Epstein–Barr virus^2^, vascular blood-brain barrier disruption^3^, microbiome-gut-brain axis alterations^4^, oxidative damage to macromolecules^5^, and mitochondrial dysfunction^6^.

Mitochondrial dysfunction (MD) is involved in the pathogenesis of multiple diseases, including cardiovascular, neurodegenerative, metabolic, and oncologic disorders^7^. Mitochondria are essential for cellular metabolism, ATP production, calcium regulation, and reactive oxygen species generation. Neurons’ high energy demands increase vulnerability to mitochondrial damage, leading to inadequate ATP production, calcium dysregulation, oxidative damage, and neurodegeneration^6^.

MD contributes to MS pathogenesis and may represent a systemic process beyond the CNS alone. In immune cells from patients with MS (PwMS), impaired mitochondrial maturation and function promote proinflammatory cytokine production, demyelination, and neurodegeneration^8^. Considering that fatigue and muscle weakness are prominent MS symptoms, skeletal muscle MD may contribute to impaired mobility in PwMS^9^.

Impaired mitochondrial function in skeletal muscle has been reported in patients with longstanding MS^9–12^. While lower physical activity, altered neuromuscular activation, and changes in muscle fiber profile (e.g., a relative reduction in oxidative type I fibers) are recognized contributors to muscle dysfunction in chronic MS, it remains unclear whether mitochondrial impairment is already present at disease onset and contributes to early clinical manifestations, or whether it develops secondarily as a consequence of disease progression and physical inactivity.

Metabolic dysfunction mediated by impaired insulin function is increasingly linked to MS^13^, contributing to cognitive impairment and neurodegeneration^14,15^. Patients with early MS show decreased insulin sensitivity with compensatory hyperinsulinemia^16^, suggesting early metabolic derangement in skeletal muscle. Insulin resistance and MD are interconnected via oxidative stress, which induces oxidative damage, interfere with insulin signaling, and further compromise mitochondrial function^17^.

Chronic inflammation, oxidative damage, and MD further contribute to the development of insulin resistance and neurodegeneration^17^. Incretins, particularly glucagon-like peptide (GLP-1), regulate postprandial insulin secretion, maintain insulin sensitivity, and influence cognition. GLP-1 also acts within the CNS, where GLP-1 receptors are widely expressed. The neuroprotective effects of GLP-1 receptor activation are thought to involve increased intracellular cAMP and respective downstream signaling pathways, promoting cell survival, inhibiting apoptosis, activating calcium channels, and supporting cellular growth, repair, and regeneration^18^. Additional anti-inflammatory effects include reducing nuclear factor-κB activation and enhancing AMP-activated protein kinase signaling, while also promoting the differentiation of oligodendrocyte progenitor cells and myelination. Collectively, these properties have made GLP-1 receptor activation a promising therapeutic target in MS^19,20^.

Beyond its classical role in systemic glucose metabolism, GLP-1 has been suggested to influence cellular energy metabolism and mitochondrial function in peripheral tissues, including skeletal muscle, linking systemic metabolic regulation to muscle bioenergetics^21^. This is particularly relevant in the context of MS, where both systemic metabolic alterations and skeletal muscle energetic dysfunction have been reported^9,16^. GLP-1 receptor activation also exerts systemic metabolic effects, particularly improving skeletal muscle insulin sensitivity, enhancing substrate utilization, and potentially influencing mitochondrial function. It is currently used in the treatment of type 2 diabetes mellitus.

**Fig. 1:**
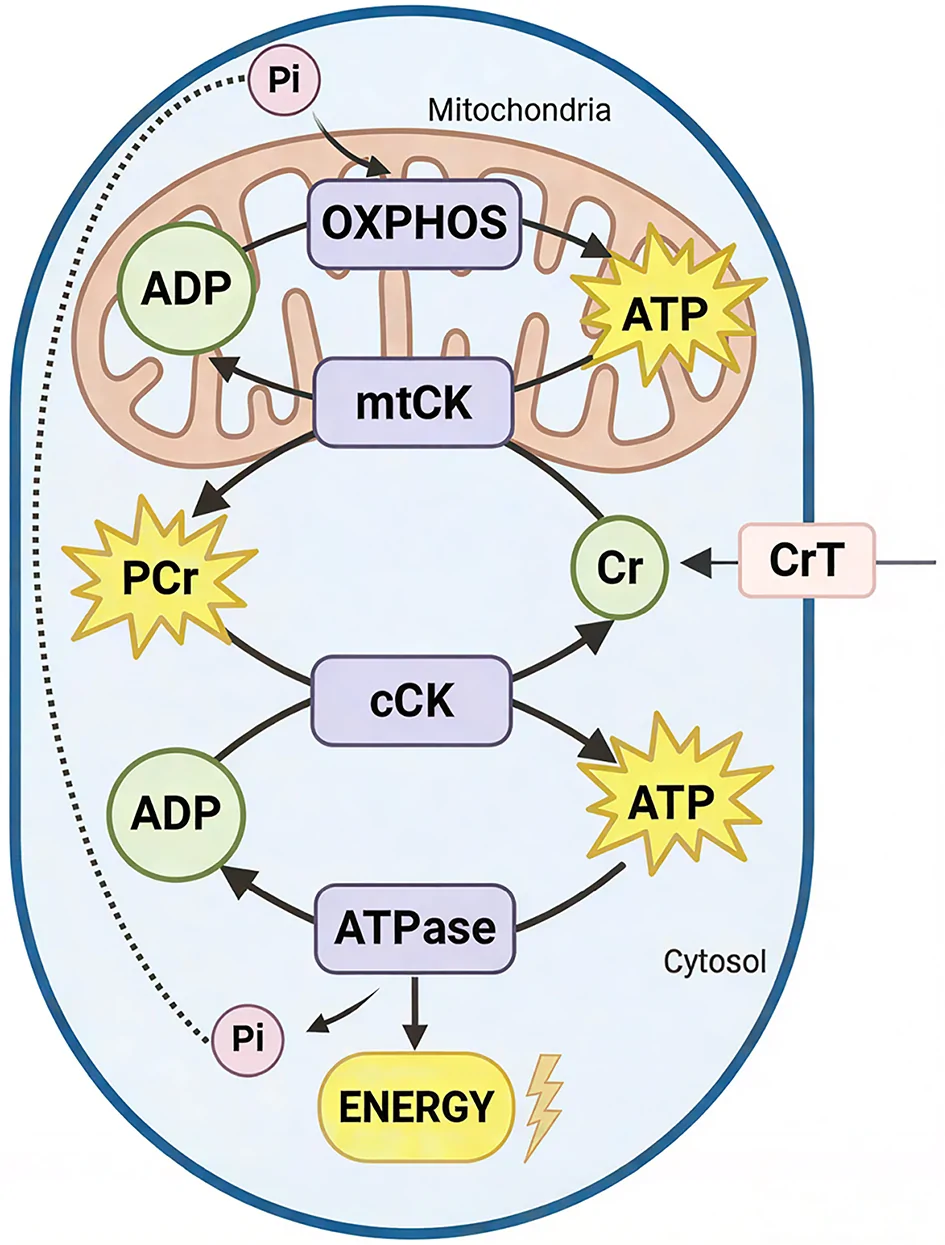
Simplified schematic illustration of the phosphocreatine (PCr) shuttle: In mitochondria, ATP produced by oxidative phosphorylation (OXPHOS) donates its high-energy phosphate to creatine (Cr) via mitochondrial creatine kinase (mtCK), generating PCr and ADP. Cr enters the cell through the creatine transporter (CrT). PCr then diffuses into the cytosol, where cytosolic creatine kinase (cCK) regenerates ATP from ADP for local energy-demanding processes (e.g., contraction, ion pumps, and channel activity). Inorganic phosphate (Pi), produced mainly by ATP hydrolysis (ATPase activity) during cellular work, is transported back into mitochondria and used by ATP synthase in OXPHOS to synthesize new ATP. Adapted and modified from^22^. Figure created with BioRender.com (free version).

Phosphocreatine (PCr) maintains ATP for skeletal muscle metabolism, membrane potential, and contraction. ATP hydrolysis (ATP → ADP + Pi + energy) releases energy for muscle contraction, with Pi subsequently used in mitochondrial oxidative phosphorylation to regenerate ATP (Fig. 1)^22^.

To address the unresolved question of early skeletal muscle mitochondrial dysfunction in MS, we applied a combined static and dynamic ^1^H/^31^P magnetic resonance spectroscopy (MRS) approach, which, to our knowledge, has not been previously applied in treatment-naïve PwMS. ¹H-MRS quantifies metabolites such as carnosine, acetylcarnitine, and intramyocellular lipids (IMCL), reflecting metabolic flexibility, oxidative stress, and muscle fiber composition^23,24^. ³¹P-MRS provides established insights into high-energy phosphate metabolism and mitochondrial capacity in vivo, indicating cell growth, tissue remodeling, and disease activity across multiple disorders^25,26^. By integrating both modalities, this approach non-invasively captures both static biochemical composition and dynamic functional responses, allowing detection of subtle muscle energetic changes that may precede clinical symptoms, and enabling the study of early metabolic and neuromuscular alterations in MS.

The aim of our study was to compare skeletal muscle metabolism in recently diagnosed, treatment-naïve patients with MS and healthy controls using combined 7 T static and dynamic ^1^H/^31^P-MRS measurements of the gastrocnemius muscle. We further investigated whether alterations in mitochondrial function and key metabolite content are already present at an early stage of the disease. By integrating complementary metabolic and bioenergetic measurements, we also explored the relationship between muscle energetics and systemic metabolic regulation, including incretin (GLP-1) response.

## Subjects and methods

This observational matched case-control study is reported in accordance with the STROBE guidelines. Twenty-six treatment-naïve patients with suspected MS were admitted to the MS center at the 1st Department of Neurology, Faculty of Medicine, Comenius University and University Hospital, Bratislava, Slovakia. All these patients underwent the examinations described below during their first hospital stay. The examination results of 16 patients, in whom the diagnosis of relapsing-remitting form of MS was confirmed according to the McDonald criteria^27^ after approximately 24 months of clinical follow-up, were used for statistical evaluation^28^. Fourteen sex-, age-, and BMI-matched healthy subjects, recruited from the employees of the 1st Department of Neurology and Biomedical Research Center, underwent the same examinations and served as controls. Of all the study participants, 9 PwMS and 9 matched healthy controls agreed to participate in the MRS investigation, which was performed within 6 weeks after initial research examinations. After a detailed explanation, written informed consent was obtained from all participants before the research examinations. The study was approved by the Ethics Committee of the Faculty of Medicine, Comenius University and University Hospital in Bratislava, Slovakia (reference number 110/2016) and by the Ethics Committee of the Bratislava Self-Governing Region, Bratislava, Slovakia (reference number 00581/2017/HF). The study was registered at ClinicalTrials.gov (NCT 03052595). The study was also approved by the Ethics Committee of the Medical University of Vienna, Austria (EK 754/2011). The study was performed following the ethical principles of the 1964 Declaration of Helsinki and its later revisions.

Inclusion criteria for all study participants were: (a) no history of cardiovascular, metabolic, endocrine, hepatic, or renal disease, malignancy, acute or chronic infection; (b) non-smokers; (c) women not pregnant or breastfeeding; and (d) not currently on any medication.

Participants were asked to abstain from food, intense physical activity, smoking, caffeine, alcohol, and any medication for 12 h prior to the research examination. Anthropometric parameters were determined with quadrupedal bioimpedance (BF-511, Omron, Kyoto, Japan), blood pressure was recorded after at least 15 min of rest. An intravenous catheter was inserted into the cubital vein, and the baseline blood samples were collected after at least 15 min of stabilization.

An oral glucose tolerance test (OGTT) was performed to measure glucose metabolism. After baseline sampling, participants ingested 75 g of glucose dissolved in 250 ml of water. Blood samples were collected 15, 30, 45, 60, 90, and 120 min after glucose ingestion. EDTA plasma aliquots were stored at -70 °C until analysis.

Plasma glucose concentrations and fasting serum total cholesterol, HDL, LDL, and triglyceride levels were analyzed in a certified hospital laboratory using an autoanalyzer (Siemens Healthcare Diagnostics Inc., Tarrytown, NY, USA) by standard procedure using enzymatic kits (Roche Diagnostics, Lewes, UK). ELISA kits were used to measure plasma concentrations of insulin (ALPCO Diagnostics, Salem, NH, USA), glucose-dependent insulinotropic polypeptide (EMD Millipore Corp., Billerica, MA, USA), and GLP-1 (Mercodia AB, Uppsala, Sweden).

Insulin sensitivity/resistance indices (ISI) were calculated using fasting and OGTT-derived glucose and insulin concentrations as described previously^29^. Insulin and GLP-1 responses to oral glucose load were calculated as Area Under the Curve (AUC) using the trapezoidal rule.

The level of physical activity was assessed using the Slovak version of the Lagerros Energy Expenditure Questionnaire (EEQ) to quantify total energy expenditure during an average weekday^30^.

**Fig. 2:**
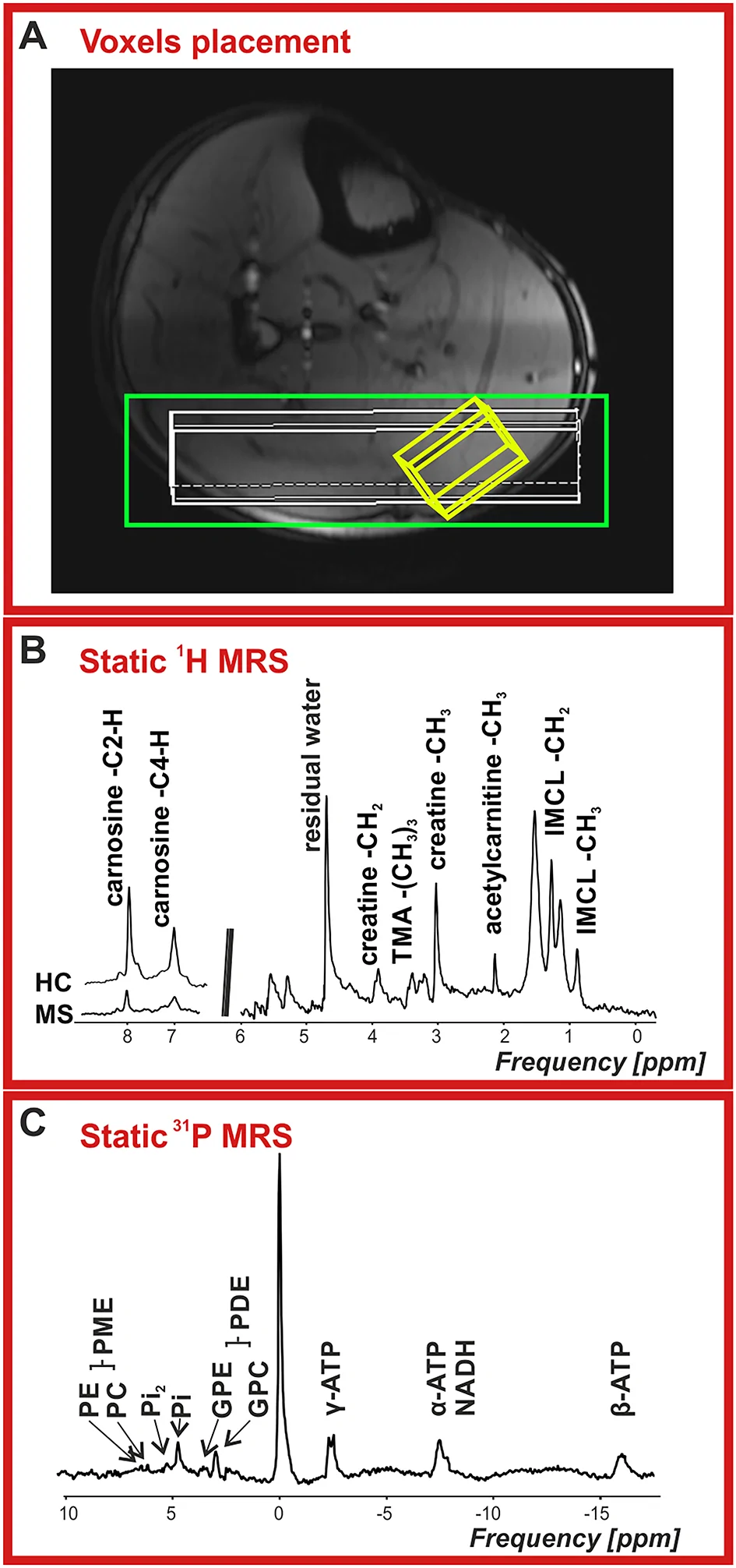
Schematic representation and spectra of skeletal muscle magnetic resonance spectroscopy (MRS) in patients with multiple sclerosis. **A** Voxel placements: Localizer image of a human calf indicating the localization of volumes of interest (VOIs). White: slice selected for ^31^P MRS, yellow: 3D-localized voxel for ^1^H MRS, green: shim volume. **B** Static ^1^H MRS: A representative ^1^H-MRS spectra acquired from the gastrocnemius lateralis muscle. In the carnosine spectral lines at 7 and 8 ppm, we can observe the difference between a healthy control (HC) and a patient with multiple sclerosis (MS). Rest of the spectra shows residual water at 4.7 ppm, creatines at 3.03 and 3.9 ppm, the trimethyl ammonium (TMA) groups of carnitine at 3.2 ppm, acetylcarnitine at 2.13 ppm, and intramyocellular lipid (IMCL) resonance lines at 0.9 and 1.3 ppm (CH_2_ group). For better visualization, a 2-Hz Lorentzian apodization filter was applied to reduce noise in the spectra. **C** Static ^31^P MRS: A representative highly spectrally resolved ^31^P-MRS spectra. For better visualization, a 2-Hz Lorentzian apodization filter was applied to reduce noise in the spectra.

All MR measurements were performed within a single visit, on a 7 T whole-body MR scanner (Magnetom; Siemens Healthineers, Erlangen, Germany) in the High Field MR Center, Department of Biomedical Imaging and Image-Guided Therapy, Medical University of Vienna, Austria. A 28-channel knee coil (QED, Mayfield Village, OH, USA) was used to acquire ^1^H-MR spectra, and a dual-tuned (^31^P/^1^H) circular surface coil (10 cm diameter, Rapid Biomedical, Rimpar, Germany) was used to acquire ^31^P-MR spectra from the calf muscle of the right leg (Fig. 2A).

### ^1^H MRS

Participants were in the supine position, with their right calf placed inside the 28-channel knee coil for ^1^H-MRS data acquisition. Based on T_1_-weighted, multi-slice localizer images, the volumes of interest (VOI) for acetylcarnitine and carnosine (40 × 30 × 12 mm^3^) and IMCL (15 × 30 × 12 mm^3^) were selected in the gastrocnemius muscle using a STimulated Echo Acquisition Model (STEAM) localization sequence. Localized shimming was performed manually, on the adjustment volume matching the VOI, after automatic field-map acquisition based on gradient recalled, double-echo field-map acquisition (GRE-SHIM, Siemens Healthineers, Erlangen, Germany). The final linewidth of the water signal was in the range of 28–38 Hz in the magnitude mode.

The MR signals of acetylcarnitine^23^, carnosine^31^, and IMCL^25^ were measured with the following parameters: acetylcarnitine (repetition time (TR) = 2000 ms, echo time (TE) = 350 ms, spectral bandwidth = 3 kHz, number of averages (NA) = 128, and delta frequency relative to water (Δ) = -2.5 ppm); carnosine (TR = 5000 ms, TE = 20 ms, spectral bandwidth = 3 kHz, NA = 64, Δ = 2.8 ppm); and IMCL (repetition time (TR) = 2000 ms, echo time (TE) = 20 ms, spectral bandwidth = 3 kHz, number of averages (NA) = 32, and delta frequency relative to water (Δ) = -3.4 ppm).

For acquisition of a concentration reference, the water signal was measured with a TR = 2000 ms, TE = 20 ms, NA = 1, and delta frequency = 0 ppm and metabolite concentrations were calculated applying appropriate T1- and T2- corrections^32^.

### ^31^P MRS

Following the proton magnetic resonance spectroscopy, the participants were positioned onto an MR-compatible ergometer (Trispect, Ergospect, Innsbruck, Austria) with the right calf placed over the ^31^P/^1^H surface coil (10 cm, Rapid Biomedical, Rimpar, Germany) for ^31^P-MRS.

Depth-resolved in vivo spectroscopy (DRESS) localization sequence was used in all static and dynamic ^31^P-MRS experiments, and the shimming was performed manually. The radiofrequency transmit voltage was adjusted based on calibration measurements with increasing radiofrequency power and searching for the maximum of the localized PCr signal. Dynamic measurement, consisting of 2 min rest phase for acquisition of baseline data, 6 min of exercise phase (plantar flexion, one per TR (2s), at a workload of approximately 25–30% of maximal voluntary contraction assessed before MRS examination), and 6 min of recovery phase, was performed as described previously^25,33^.

All spectra analyses and parameter calculations were described previously^33^. Briefly, all the acquired spectra were fitted and analyzed using the time domain-fitting algorithm AMARES in jMRUI. Resonance lines of metabolites related to cellular energy metabolism and membrane phospholipid metabolism were fitted as single Lorentzians. During recovery phase, the PCr recovery curve was fitted mono-exponentially to determine the time constant of PCr recovery rate. Intramyocellular pH at rest and post-exercise was calculated from the chemical shift of PCr and Pi signals using the modified Henderson–Hasselbalch equation. Two participants (1 patient and 1 control) were excluded from certain dynamic calculations due to an inappropriate workload setting during examination. Because the workload resistance was not correctly adjusted, the PCr depletion during exercise was insufficient, resulting in a poor goodness-of-fit (R² ≈ 0.6, instead of the typical 0.8–0.99).

IBM SPSS Statistics version 25 (SPSS Inc., Chicago, IL, USA) was used for statistical analyses. Group differences in mean values were analyzed by Student’s *t* test or the Mann–Whitney *U* test, pre-post differences within groups were analyzed by paired t test or the Wilcoxon signed-rank test, depending on the normality of the data distributions as assessed by the Shapiro–Wilk test. Changes in metabolic parameters during the OGTT were analyzed using the general linear mixed model for repeated measures. Metabolic testing results were correlated with MRS data using Pearson’s or Spearman’s correlation, depending on data distribution. All values are expressed as mean ± standard deviation (SD) unless stated otherwise. A *p* value of less than 0.05 was considered statistically significant. No formal sensitivity analyses were performed.

## Results

Anthropometric characteristics, level of daily physical activity, and baseline metabolic parameters were comparable between PwMS and healthy controls, including the selected subgroups undergoing MRS examination (Table 1). All PwMS had an Expanded Disability Status Scale (EDSS) < 2, indicating a minimal disability.

**Table 1:** Clinical characteristics and metabolic parameters of PwMS and matched healthy controls.

Parameter	Total		MRS group	
	PwMS (*n* = 16)	Controls (*n* = 14)	PwMS (*n* = 9)	Controls (*n* = 9)
Sex (M/F)	6/10	7/7	3/6	3/6
Age (years)	30.1 ± 7.3	31.4 ± 8.9	29.2 ± 6.9	29.7 ± 8.3
BMI (kg/m²)	22.1 ± 3.4	23.0 ± 2.8	23.0 ± 2.3	23.0 ± 2.7
Fat mass (%)	25.6 ± 10.6	24.9 ± 7.6	23.3 ± 12.5	25.7 ± 8.5
Fat-free mass (kg)	49.8 ± 11.6	54.4 ± 12.9	49.2 ± 12.4	49.6 ± 11.5
Muscle mass (kg)	21.7 ± 7.2	25.0 ± 7.6	21.6 ± 7.8	22.2 ± 7.1
Waist circumference (cm)	81 ± 10	82 ± 9	79 ± 10	78 ± 6
Systolic BP (mmHg)	112 ± 8	117 ± 12	109 ± 9	113 ± 12
Diastolic BP (mmHg)	71 ± 7	72 ± 9	71 ± 8	70 ± 9
Heart rate (min^−1^)	70 ± 10	64 ± 9	71 ± 10	66 ± 6
Energy expenditure (kCal/24 h)	2655 ± 780	2997 ± 770	2637 ± 939	2615 ± 363
Total cholesterol (mmol l^−1^)	4.24 ± 0.67	4.07 ± 0.62	4.17 ± 0.70	4.34 ± 0.53
HDL cholesterol (mmol l^−1^)	1.25 ± 0.29	1.29 ± 0.27	1.31 ± 0.33	1.38 ± 0.30
LDL cholesterol (mmol l^−1^)	2.77 ± 0.54	2.55 ± 0.52	2.67 ± 0.60	2.70 ± 0.54
VLDL cholesterol (mmol l^−1^)	0.34 ± 0.10	0.37 ± 0.16	0.33 ± 0.10	0.34 ± 0.11
Triglycerides (mmol l^−1^)	0.75 ± 0.23	0.82 ± 0.35	0.73 ± 0.23	0.74 ± 0.24
Glucose 0 min (mmol l^−1^)	4.84 ± 0.46	4.69 ± 0.46	4.60 ± 0.29	4.62 ± 0.53
Glucose 120 min (mmol l^−1^)	5.89 ± 1.86	5.36 ± 1.33	5.11 ± 2.08	5.52 ± 1.43
Insulin 0 min (mIU l^−1^)	6.52 ± 4.58	6.03 ± 2.54	6.85 ± 5.49	6.07 ± 2.71
Insulin 120 min (mIU l^−1^)	52.9 ± 44.3	32.1 ± 13.4	32.6 ± 19.1	37.2 ± 11.7
HOMA-IR	1.40 ± 0.95	1.28 ± 0.58	1.41 ± 1.13	1.28 ± 0.63
ISI Matsuda	7.54 ± 4.0	8.54 ± 5.64	8.60 ± 4.68	9.28 ± 7.02
ISI Cederholm	61.3 ± 17.4	65.8 ± 16.4	69.9 ± 17.8	65.7 ± 20.6
GLP-1 fasting (pmol l^−1^)	3.51 ± 2.02	3.29 ± 1.13	3.34 ± 2.22	3.25 ± 1.28
GIP fasting (pmol l^−1^)	12.4 ± 15.7	8.4 ± 3.6	13.2 ± 17.3	8.3 ± 4.5

Values are presented as mean ± SD. No statistically significant differences were observed between PwMS and Controls, including the selected subgroups undergoing MRS examination. *BMI* body mass index, *BP* blood pressure, *HDL* high density lipoprotein, *LDL* low density lipoprotein, *VLDL* very low density lipoprotein, *ISI* insulin sensitivity index, *GLP-1* glucagon-like peptide 1, *GIP* glucose-dependent insulinotropic polypeptide.

### Metabolic measurements

There were no differences in the plasma glucose, insulin, or glucose-dependent insulinotropic polypeptide concentration profiles during the OGTT, nor in the OGTT-derived parameters of insulin sensitivity/resistance between patients and controls. The data in Table 1 clearly demonstrate that the MRS-subgroup is representative of the entire investigated cohort.

**Fig. 3:**
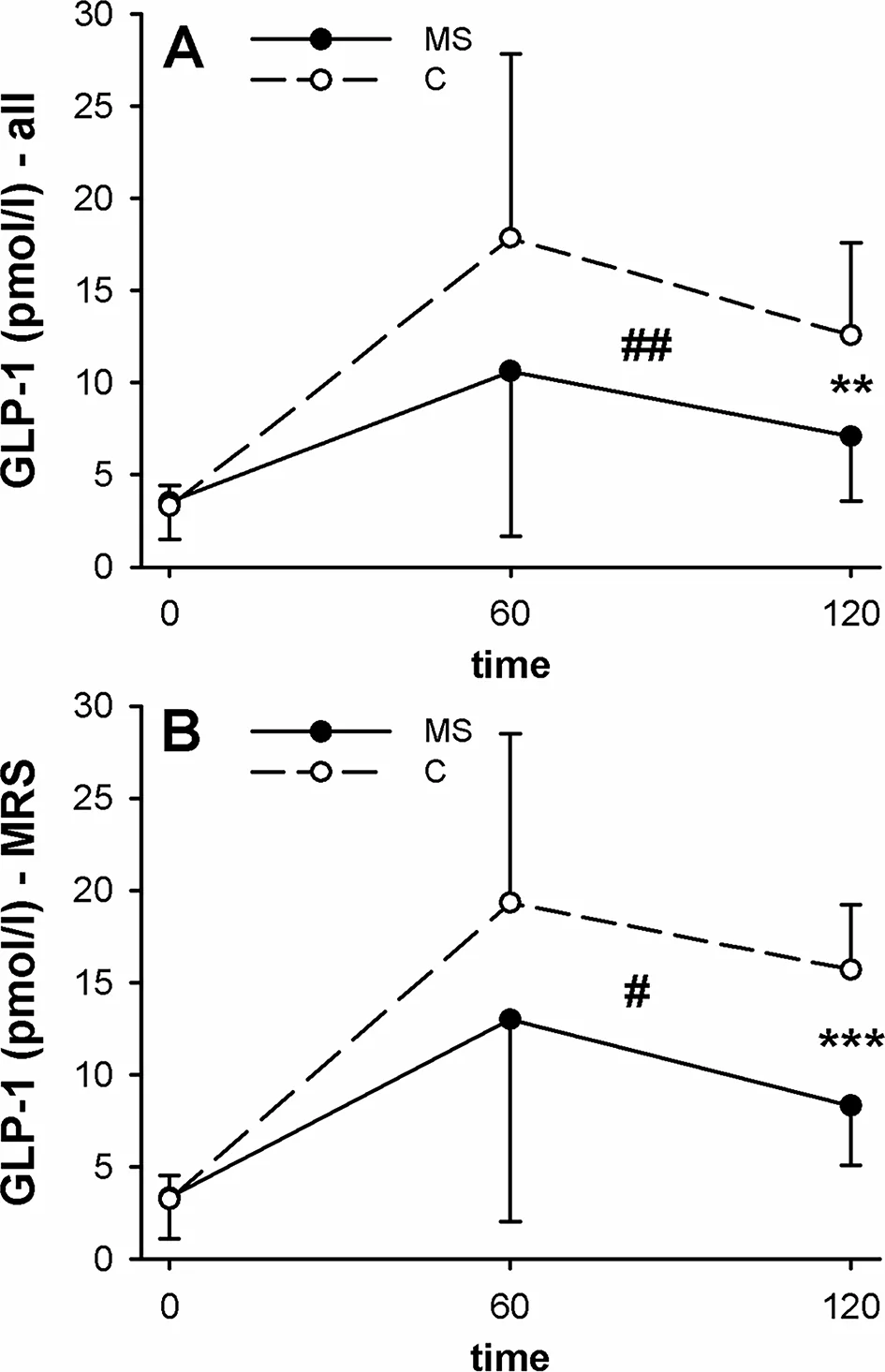
Plasma GLP-1 concentrations in response to oral glucose load in PwMS and healthy controls. **A** Time course of GLP-1 levels (mean ± SD) in all participants (PwMS (MS), *n* = 16; controls (C), *n* = 14). **B** Time course in the MRS subgroup (PwMS (MS), *n* = 9; controls (C), *n* = 9).  General linear mixed model for repeated measures was used to compare the entire GLP-1 response between groups (## *p* < 0.01; # *p* < 0.05). Tukey’s post-hoc test was applied for comparisons at individual time points (*** *p* < 0.001; ** *p* < 0.01).

The GLP-1 response to oral glucose load expressed as AUC was lower in PwMS (954 ± 600 pmol·min/l vs. 1545 ± 657 pmol·min/l; *p* = 0.018). A mixed repeated measures general linear model evaluated the main and interaction effects of diagnosis and OGTT on GLP-1 plasma levels. As expected, GLP-1 increased after oral glucose load (F(2,54) = 26.853, *p* < 0.0005), which was also evident in the MRS-subgroup (F(2,30) = 20.460, *p* < 0.0005). There was a main effect of diagnosis (F(1,27) = 8.009, *p* = 0.009) on GLP-1 response to OGTT, also present in the smaller MRS-subgroup (F(1,15) = 5.596, *p* = 0.032), with average GLP-1 concentrations being significantly lower in PwMS (Fig. 3A, B). The interaction between diagnosis and OGTT did not reach statistical significance in the entire group (*p* = 0.062), nor in the MRS subgroup (*p* = 0.181).

### MRS measurements

Whole-body MRS measurements at 7 T were performed in 9 PwMS and 9 age-, sex-, and BMI-matched healthy controls (Table 2).

**Table 2:** Skeletal muscle metabolites in PwMS and healthy controls measured by 7 T ^1^H and ^31^P MRS.

	PwMS (*n* = 9)	Controls (*n* = 9)	*p*
Static ^1^H-MRS parameters			
Acetyl-carnitine (mmol kg^-1^ ww)	2.94 ± 1.88	5.53 ± 3.53	0.074
Carnosine 8 ppm (mmol kg^-1^ ww)	**4.26 ± 2.07**	**6.38 ± 1.87**	**0.044**
IMCL CH_2_	0.34 ± 0.27	0.42 ± 0.34	0.577
*Static* ^*31*^*P-MRS parameters*			
PME (mmol l^-1^) static	0.30 ± 0.08	0.37 ± 0.24	0.424
PDE (mmol l^-1^) static	3.13 ± 0.74	3.13 ± 0.60	0.997
PC (mmol l^−1^) static	0.15 ± 0.07	0.20 ± 0.16	0.433
PE (mmol l^−1^) static	0.14 ± 0.10	0.17 ± 0.12	0.669
GPC (mmol l^−1^) static	2.35 ± 0.70	2.37 ± 0.52	0.947
GPE (mmol l^−1^) static	0.78 ± 0.46	0.76 ± 0.19	0.913
Pi_2_/Pi	0.082 ± 0.061	0.101 ± 0.039	0.434
*Dynamic* ^*31*^*P-MRS parameters*			
pH rest	7.05 ± 0.02	7.05 ± 0.02	0.580
pH end of exercise	6.99 ± 0.07	6.98 ± 0.08	0.798
Pi (mmol l^-1^) rest	**3.48 ± 0.61**	**2.79 ± 0.40**	**0.012**
Pi (mmol l^-1^) end of exercise	9.96 ± 2.97	8.78 ± 3.49	0.449
PCr (mmol l^-1^) rest	28.8 ± 5.2	26.8 ± 4.1	0.378
PCr (mmol l^-1^) end of exercise	22.1 ± 4.6	21.2 ± 5.0	0.673
PCr (mmol.l^-1^) recovery	30.1 ± 4.7	26.8 ± 3.7	0.123
PCr/Pi ratio rest	8.33 ± 0.77	9.81 ± 2.02	0.056
PCr drop (%)	27.90 ± 13.91	22.07 ± 20.85	0.521
τ_PCr_ (s)	**40.7 ± 7.5**	**30.2 ± 9.9**	**0.031**

Values are presented as mean ± SD. *IMCL* intramyocellular lipids, *PDE* phosphodiester, *PME* phosphomonoester, *PC* phosphocholine, *PE* phosphoethanolamine, *GPC* glycerophosphocholine, *GPE* glycerophosphoethanolamine, *Pi* inorganic phosphate, *Pi*_*2*_*/Pi* ratio of a secondary (mainly mitochondrial) to the cytosolic inorganic phosphate pools, *PCr* phosphocreatine, *τ*_*PCr*_ time constant of PCr resynthesis, *ww* wet weight. Bold type indicates statistically significant diff erences (p<0.05).

#### Static ^1^H-MRS parameters

PwMS had significantly lower concentration of carnosine in the gastrocnemius muscle (*p* = 0.044), reaching about 66% of the level in controls (Table 2). In the entire study subgroup, lower carnosine levels were associated with higher resting Pi levels (*r* = − 0.639, *p* = 0.006); higher carnosine levels were associated with greater PCr drop in PwMS only (*r* = 0.740, *p* = 0.036). Correlations trending towards significance were observed between the content of carnosine and fat-free mass (*r* = 0.646, *p* = 0.060), and carnosine and muscle mass (*r* = 0.619, *p* = 0.075), again in PwMS only.

Lower acetylcarnitine levels were observed in PwMS compared to controls, although the difference did not reach statistical significance (*p* = 0.074). In the whole subgroup and in PwMS, higher acetylcarnitine was associated with faster PCr recovery (*r* = − 0.698, *p* = 0.004; *r* = − 0.727, *p* = 0.041, respectively). IMCL content was comparable between groups (Table 2). In PwMS, higher IMCL CH2 levels were associated with greater fat-free mass (*r* = 0.678, *p* = 0.045), and IMCL tended to increase with muscle mass (*r* = 0.637, *p* = 0.065).

An exemplary spectrum of ^1^H-MRS is shown in Fig. 2B.

#### Static ^31^P-MRS measurements

No substantial differences were found in phosphomonoester (or its components, phosphocholine and phosphoethanolamine) or in phosphodiester (or its components, glycerophosphocholine and glycerophosphoethanolamine) content between PwMS and controls (Table 2). The Pi_2_/Pi ratio, associated with mitochondrial function and training status, was not lower in PwMS. Within-group associations were observed: in controls, higher body fat percentage was associated with lower phosphomonoester content (*r* = − 0.714, *p* = 0.031), while phosphodiester content was associated with higher LDL cholesterol (*r* = 0.697, *p* = 0.037) and higher Pi_2_/Pi ratio (*r* = 0.688, *p* = 0.041). In PwMS, higher phosphodiester levels were associated with higher AUC insulin (*r* = 0.741, *p* = 0.022) and higher resting pH (*r* = 0.822, *p* = 0.007).

An exemplary spectrum of ^31^P-MRS is shown in Fig. 2C.

#### Dynamic ^31^P-MRS measurements

**Fig. 4:**
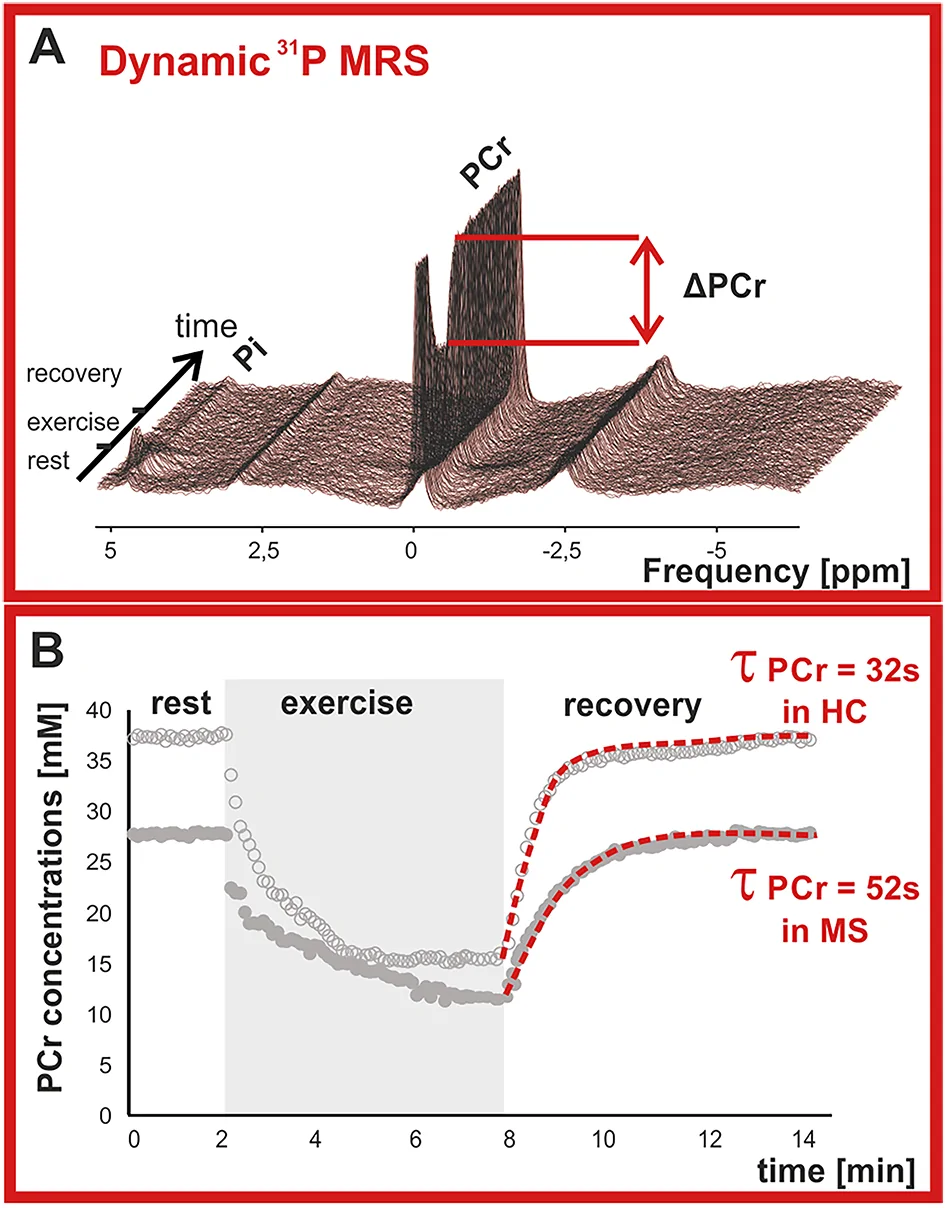
Schematic representation of dynamic spectra of skeletal muscle magnetic resonance spectroscopy (MRS) in patients with multiple sclerosis. **A** Dynamic ^31^P MRS: Time course of a ^31^P MR spectra during a plantar flexion exercise with depicted depletion of the PCr signal and its subsequent re-synthesis during the recovery period. **B** Representative time series of PCr evolutions during 2 min at rest, 6 min of plantar flexion exercise, and 6 min of recovery from the gastrocnemius muscle of one healthy control (HC) and one patient with multiple sclerosis (MS) are shown. Dashed red lines—fit of time constant of PCr resynthesis (τ_PCr_) are depicted.

Pre-exercise Pi measured at rest during the dynamic ³¹P-MRS protocol was higher in PwMS (*p* = 0.012), the corresponding PCr/Pi ratio at rest lower without statistical significance (*p* = 0.056), suggesting subtle alterations in muscle energy metabolism. No group differences were observed at the end of exercise or during recovery. An exemplary spectrum of dynamic ^31^P-MRS is shown in Fig. 4A.

During exercise, pH decreased significantly in both PwMS (*p* = 0.014) and controls (*p* = 0.004), with no difference between groups at rest or at the end of exercise (Table 2). However, the magnitude of the decrease was small (ΔpH = 0.07), remaining well within the physiological limits and unlikely to affect muscle metabolic function. Interestingly, higher resting pH was associated with greater muscle mass in controls only (*r* = 0.854, *p* = 0.003).

In PwMS, higher PCr levels at rest, during exercise, and during recovery were associated with higher AUC GLP-1 in PwMS (*r* = 0.834, *p* = 0.005; *r* = 0.681, *p* = 0.043, and *r* = 0.730, *p* = 0.026, respectively), whereas no such associations were observed in controls.

The time constant of PCr recovery after exercise was longer in PwMS (40.7 ± 7.5 s), reaching about 135% of that in controls (30.2 ± 9.9 s, *p* = 0.031), indicating slower post-exercise PCr resynthesis (Fig. 4B). In controls, PCr recovery time increased with AUC insulin (*r* = 0.843, *p* = 0.009) and HOMA-IR (*r* = 0.774, *p* = 0.024), whereas this relationship was absent in PwMS.

## Discussion

Our study aimed to detect potential metabolic differences in skeletal muscles between recently diagnosed, yet treatment-naïve PwMS and healthy controls, in relation to whole-body metabolic status.

### Metabolic status

The overall metabolic status of PwMS was comparable to that of healthy controls, with the only difference being a diminished GLP-1 response to oral glucose load in PwMS, despite comparable insulin sensitivity. GLP-1, a gut peptide secreted by enteroendocrine L cells in response to ingested nutrients, enhances glucose-stimulated insulin release, inhibits glucagon secretion, reduces appetite, modulates gastrointestinal function, and has proposed neurotropic and neuroprotective effects^18,19^. Given the proposed role of peripheral GLP-1 in the CNS, it can be hypothesized that a reduced influx of this incretin hormone could potentially impair axonal and neuronal regeneration in MS. Furthermore, treatment with GLP-1 receptor agonists may represent a plausible therapeutic strategy to support neuronal and axonal repair in MS lesions. Impaired GLP-1 secretion has been reported in type 2 diabetes, prediabetes, and obesity, suggesting that impaired incretin function may contribute to insulin resistance^34^. While GLP-1 is well known for its crucial role in promoting insulin secretion, experimental evidence suggests that insulin itself can stimulate GLP-1 secretion, potentially contributing to a feedback interaction that may be relevant to insulin resistance^35^. Our finding of a decreased glucose-stimulated GLP-1 response in newly diagnosed, yet treatment-naïve PwMS suggests that alterations in incretin physiology may be present at disease onset, potentially preceding overt metabolic dysfunction and contributing to systemic metabolic dysregulation in MS.

### Muscle MRS-derived parameters

Static ^1^H-MRS revealed lower carnosine levels in the gastrocnemius muscle of recently diagnosed, treatment-naïve PwMS compared to controls, consistent with previous HPLC-based muscle biopsy studies^36^.

Carnosine, a dipeptide of histidine and beta-alanine, is abundant in skeletal muscle, heart, and neuronal tissue. It regulates muscle pH during exercise, modulates calcium handling, and has antioxidant properties, contributing to skeletal muscle homeostasis and delaying the onset of muscle fatigue after intense physical activity^31,32^. Its concentration varies with age, sex, and muscle fiber type, with lower levels in slow-twitch (type I) than fast-twitch (type II) fibers^24^. Lower muscle carnosine levels may reflect either a shift from fast-twitch to slow-twitch muscle fibers^37^, age-related reductions in physical activity and muscle mass loss, or a decrease of carnosine content in Type II muscle fibers^25^. In our cohort, PwMS were all under 45 years, had comparable muscle mass and daily physical activity to controls, suggesting that reduced carnosine content itself, rather than the transition from fast-twitch to slow-twitch fibers, likely explains the difference.

Carnosine supplementation has been proposed to reduce low-grade inflammation and oxidative damage^38^, but clinical studies in prediabetes and diabetes have shown modest or negligible benefits for cardiovascular and cardiometabolic risk, musculoskeletal health, inflammation, and biomarkers of oxidative damage^39^. In MS, dietary carnosine failed to improve muscle function in a rodent model^40^; positive effects in human MS studies are only modestly reported^41^.

Acetylcarnitine levels tended to be lower in PwMS, but the difference was not statistically significant. Acetylcarnitine plays a role in skeletal muscle mitochondrial metabolism and may support metabolic flexibility, insulin sensitivity, and substrate utilization by buffering acetyl-CoA availability for the Krebs cycle^42^. Reduced acetylcarnitine is associated with insulin resistance, impaired mitochondrial function, and lower physical activity^23,32^. Previous studies found lower acetylcarnitine levels in overweight or sedentary individuals compared to lean, active counterparts^42^. Trials of acetyl-L-carnitine treatment for MS-related fatigue have yielded inconsistent results^43^. The reduction of both carnosine and acetylcarnitine, in the context of previous studies reporting limited efficacy of supplementation strategies, may indicate that their intramuscular content is primarily determined by intrinsic metabolic regulation rather than dietary intake alone, and may reflect a shift in intramuscular metabolic homeostasis, including altered synthesis, turnover, or utilization.

IMCL content did not differ between PwMS and controls. IMCL are small lipid droplets near mitochondria within myocytes^32^, increasing with aging, insulin resistance, obesity, and type 2 diabetes, though the “athlete’s paradox” describes higher IMCL in athletes despite high insulin sensitivity^44^. IMCL levels are influenced by insulin sensitivity, mitochondrial activity, physical activity, and diet^32^.

Phosphomonoesters participate in membrane synthesis, while phosphodiesters reflect membrane lipid breakdown^45^ and/or the dysfunction of glycerophosphocholine phosphodiesterase 1 (Gpcpd1) in aging and insulin resistance^26^. Their quantification provides insight into cellular metabolism and membrane turnover. The lack of differences between recently diagnosed PwMS and controls in our study suggests intact muscle membrane structure, likely reflecting the young age of the cohort. Similarly, Pi_2_/Pi ratio, a marker associated with mitochondrial energetic capacity and training status^46^, was comparable, suggesting relatively preserved basal muscle bioenergetics in PwMS.

PwMS often show impaired mitochondrial oxidative capacity, as demonstrated by reduced muscle oxygen utilization measured by near-infrared spectroscopy^12^ and by alterations in mitochondrial content and oxidative enzymes in skeletal muscle biopsies^47^. Findings from peripheral immune cell studies further support the presence of systemic mitochondrial alterations in MS^8^. Such mitochondrial impairment, even at rest, may slightly reduce the efficiency of ATP regeneration in skeletal muscle, increasing cytosolic Pi levels.

In our dynamic ³¹P-MRS measurements, pre-exercise Pi at rest was significantly higher in PwMS, and the corresponding PCr/Pi ratio was lower than in controls. Elevated Pi and a trend toward a reduced PCr/Pi ratio are consistent with subtle impairments in energy buffering or mitochondrial efficiency, possibly reflecting impaired ATP/PCr equilibrium or mitochondrial dysfunction in PwMS. The calculated PCr/Pi ratio at rest reflects the muscle energy reserve and mitochondrial function^48^ and typically decreases during tissue ischemia or physical activity^49^.

As already mentioned, PCr acts as a rapidly available energy reserve for ATP synthesis during muscle contraction. Prolonged post-exercise PCr recovery has been found in patients with longstanding MS^10^.

Slower PCr recovery after exercise in our PwMS indicates impaired oxidative phosphorylation and reduced mitochondrial capacity in skeletal muscle, consistent with previous reports^50^. This is in line with a prior ^31^P-MRS study demonstrating delayed PCr recovery in PwMS^11^ and with near-infrared spectroscopy-based evidence of reduced muscle mitochondrial capacity in PwMS^12^. Similar metabolic patterns (elevated Pi, lower PCr/Pi ratio at rest, and prolonged PCr recovery) have been reported in patients with mitochondrial myopathy^51^, as well as with longstanding MS (mean duration 13 years)^10^. Our findings in recently diagnosed PwMS indicate that subtle impairments in mitochondrial energy restoration are already present at the beginning of the disease course^42^. Importantly, positive correlations between PCr recovery and markers of insulin resistance (AUC insulin and HOMA-IR) seen in controls were absent in PwMS, suggesting that disease-specific factors such as neuroinflammation, reduced physical activity, or intrinsic mitochondrial dysfunction dominate muscle energetics, decoupling mitochondrial recovery from systemic insulin sensitivity. This dissociation suggests that disease-specific mechanisms, rather than classical metabolic determinants, may play a dominant role in shaping skeletal muscle energetics in recently diagnosed MS.

The key question remains open regarding the mechanisms underlying impaired skeletal muscle energetics in newly diagnosed PwMS. Processes involving mitochondrial energy transduction, including proton transfer dynamics and electron-coupled tunneling within ATP synthase, have been discussed in the literature. However, these hypotheses cannot be inferred from our data. Recent advances in deuterium-based metabolic imaging and isotope tracing have demonstrated the feasibility of probing metabolic fluxes and pathway activity in experimental settings^52–54^. These approaches suggest that combining high-field MRS with stable isotope methodologies may in future studies provide complementary insight into early metabolic alterations in neurodegeneration. Our results reflect integrated muscle bioenergetics rather than specific molecular mechanisms.

Slower PCr recovery has also been reported in overweight, sedentary, or older individuals compared with lean, active, or younger participants, reflecting the influence of age, physical activity, and fitness on mitochondrial capacity^25,42^. However, in our study, PwMS and controls demonstrated comparable habitual physical activity as assessed by the Lagerros questionnaire^30^, suggesting that reduced physical activity alone cannot explain impaired PCr recovery in PwMS. This indicates that skeletal muscle MD is present in the early phase of MS, possibly contributing to impaired muscle energetics independently of traditional metabolic risk factors.

Positive correlation of GLP-1 responses and PCr levels during the dynamic study in PwMS only may reflect a disease-specific link between skeletal muscle energetics and postprandial incretin secretion. Recent evidence suggests that GLP-1 may influence skeletal muscle mitochondrial biology, including mitochondrial morphology, density, and oxidative capacity, although current data are largely derived from experimental and preclinical studies and remain partly inconsistent^21^. In this context, our findings raise the possibility that altered GLP-1 secretion may contribute to differences in mitochondrial energy handling in skeletal muscle, particularly under conditions of increased energetic demand. This observation extends current understanding by suggesting that systemic metabolic regulation may interact with local muscle bioenergetics already in early MS. However, given the observational nature of the present study and the limited direct evidence in humans, this hypothesis remains speculative and requires confirmation in larger and mechanistic studies.

Minor but consistent decreases in intracellular pH during exercise (ΔpH ≈ 0.07) were observed in both groups, reflecting ATP hydrolysis and subtle lactate production. Although statistically significant, the changes were minor, within the normal physiological range, and unlikely to affect muscle metabolism or function^55^, indicating preserved muscle acid-base regulation and suggesting that basic metabolic buffering capacity remains intact in early MS despite detectable alterations in mitochondrial energetics.

Our findings are most applicable to early-stage patients with relapsing-remitting MS and minimal disability and may not fully generalize to patients with advanced disease, progressive MS, or substantial comorbidities. Limitations include a small number of subjects undergoing MRS examination. However, the MRS subgroup was representative of the entire cohort in terms of anthropometric and metabolic characteristics. Sample size was limited by participant availability. Findings should be confirmed in larger cohorts.

OGTT-derived indices were used instead of the euglycemic hyperinsulinemic clamp, the “gold standard” for insulin sensitivity, because OGTT more accurately mimics the physiological dynamics of glucose, insulin, and incretins and is less demanding for participants.

## Conclusion

Skeletal muscle in recently diagnosed, yet treatment-naïve PwMS exhibits signs of mitochondrial impairment, detectable only under dynamic energetic load and occurring independently of conventional metabolic risk factors. Reduced levels of carnosine and acetylcarnitine, elevated resting inorganic phosphate, and prolonged post-exercise PCr recovery in PwMS, likely reflect alterations in muscle bioenergetics and metabolic composition, potentially contributing to subtle energetic stress and muscle fatigue. Decreased GLP-1 response to an oral glucose load in patients with newly diagnosed MS may reflect early alterations in insulin action, though its clinical significance remains to be elucidated. These findings highlight skeletal muscle metabolism as potential target in MS and support further investigation of mitochondrial function as a modifiable component of disease-related metabolic dysfunction.

## Data Availability

The datasets generated during the study are available from the corresponding author upon reasonable request.

## Abbreviations

GLP-1: Glucagon-like peptide-1
HOMA-IR: Homeostasis model assessment of insulin resistance
IMCL: Intramyocellular lipids
MD: Mitochondrial dysfunction
MRS: Magnetic resonance spectroscopy
MS: Multiple sclerosis
OGTT: Oral glucose tolerance test
PCr: Phosphocreatine
Pi: Inorganic phosphate - cytosolic
PwMS: Patients with multiple sclerosis
